# Bone Resection for Isolated Ulnar Head Fracture

**DOI:** 10.1155/2017/3519146

**Published:** 2017-10-26

**Authors:** Hiromasa Akino, Shunpei Hama, Masataka Yasuda, Kenta Minato, Masahiro Miyashita

**Affiliations:** Department of Orthopaedic Surgery, Baba Memorial Hospital, 4-244 Funao-cho Higashi, Sakai, Osaka 592-8555, Japan

## Abstract

Distal ulnar fractures often occur with distal radius fractures (DRFs), and ulnar styloid fractures commonly occur in the setting of DRF. However, isolated ulnar head fractures are rare. We report a case of isolated ulnar head fracture in which we performed bone resection because the ulnar head bone fragment fractured when internal screw fixation was attempted. His outcome at 18 months postoperatively was considered excellent. However, we do not advocate bone resection other than failure of fixation and the difficult case to perform internal fixation. Longer follow-up would be needed because bone resection might lead to osteoarthritis of the distal radioulnar joint in the future.

## 1. Introduction

Distal ulnar fractures often occur with distal radius fractures (DRFs), and ulnar styloid fractures commonly occur in the setting of DRF. However, isolated ulnar head fractures are rare, and most cases in the literature have been treated with internal screw fixation. We performed bone resection for an isolated ulnar head fracture, which caused supination pain of the forearm.

## 2. Case Report

**Figure 1:**
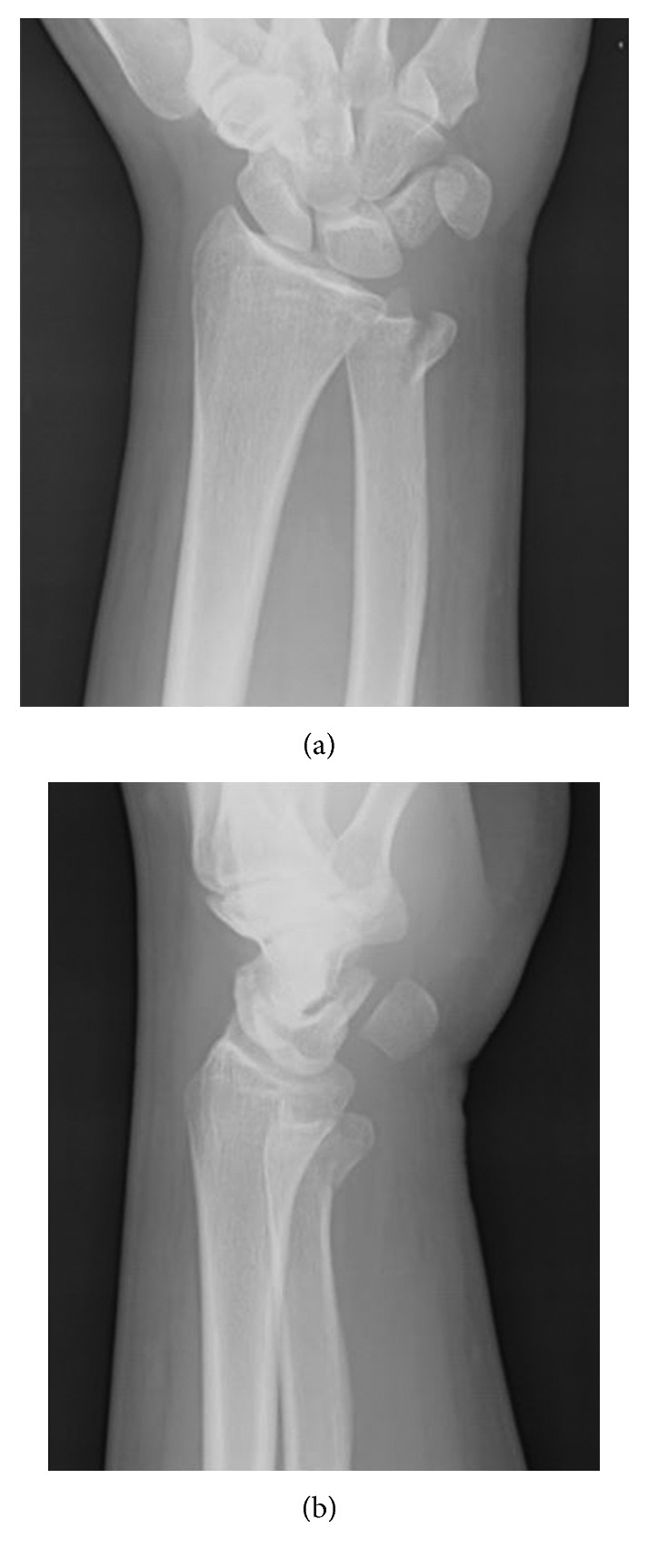
Preoperative radiographs. (a) Anterior-posterior view and (b) lateral view.

**Figure 2:**
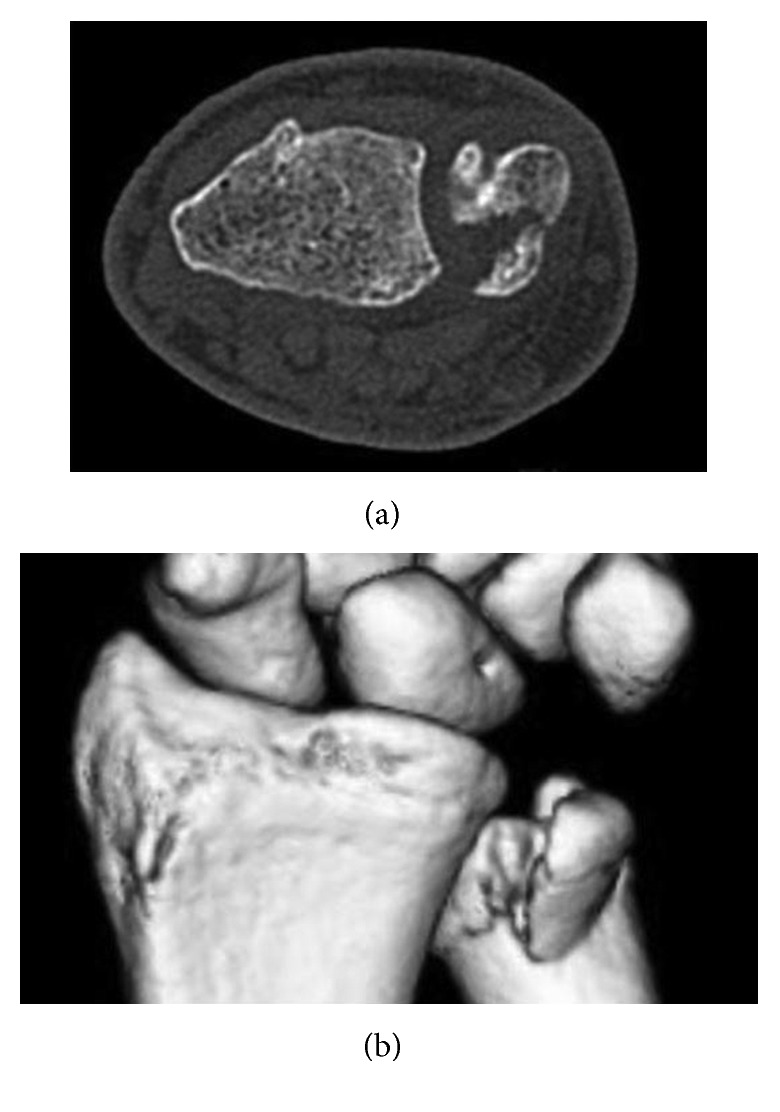
Preoperative CT in the supinated position. (a) Axial view and (b) 3D reconstruction.

**Figure 3:**
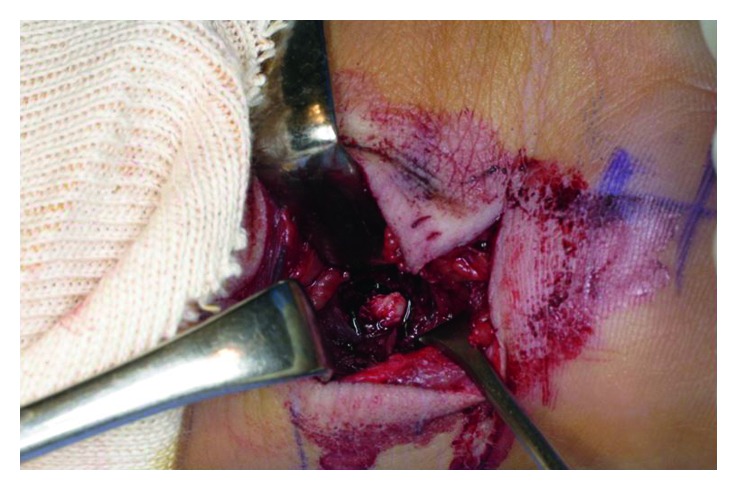
An intraoperative photograph of the wrist. Right side is distal. We swerved the ulnar nerve and the flexor digitorum profundus of the little finger to radial side and opened the capsule. Then, we could see the bone fragment from the volar incision.

**Figure 4:**
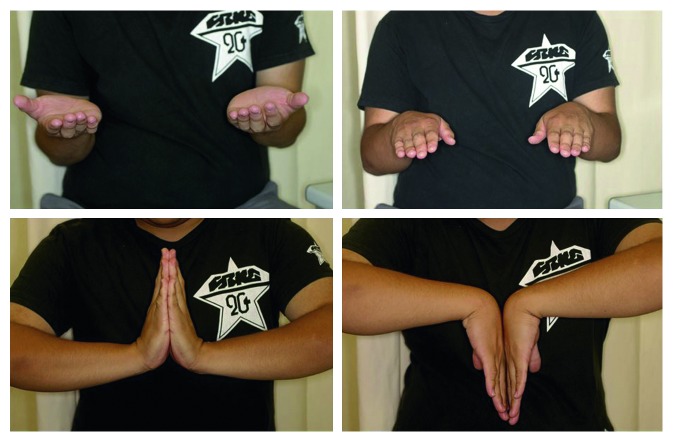
Photographs at 18 months postoperatively. The range of motion was equal to the contralateral side.

**Figure 5:**
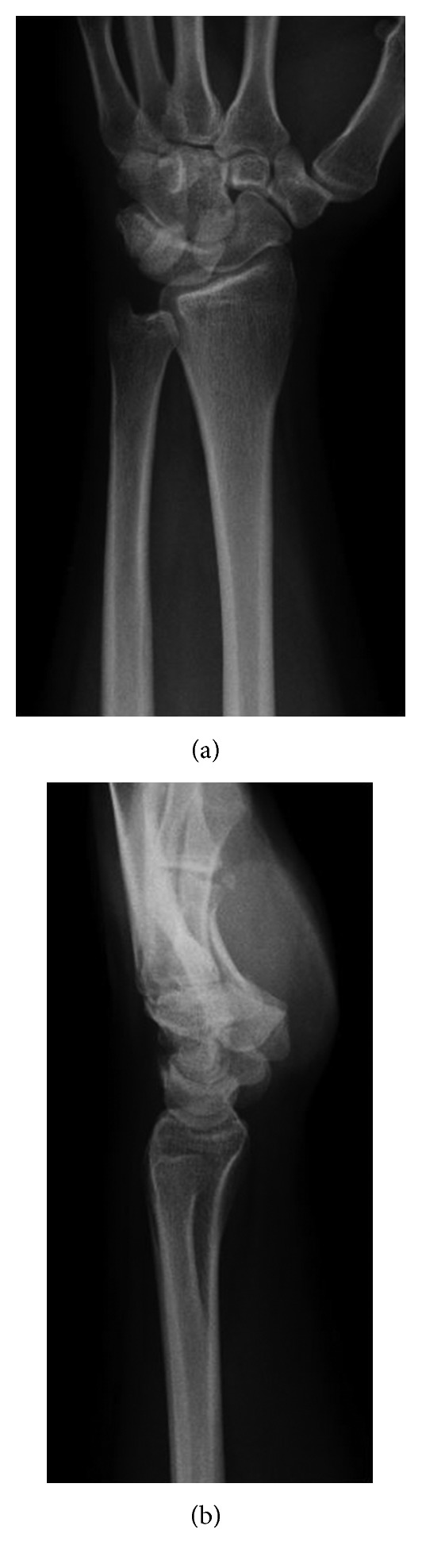
Radiographs at 18 months postoperatively. (a) Posterior-anterior view and (b) lateral view.

**Figure 6:**
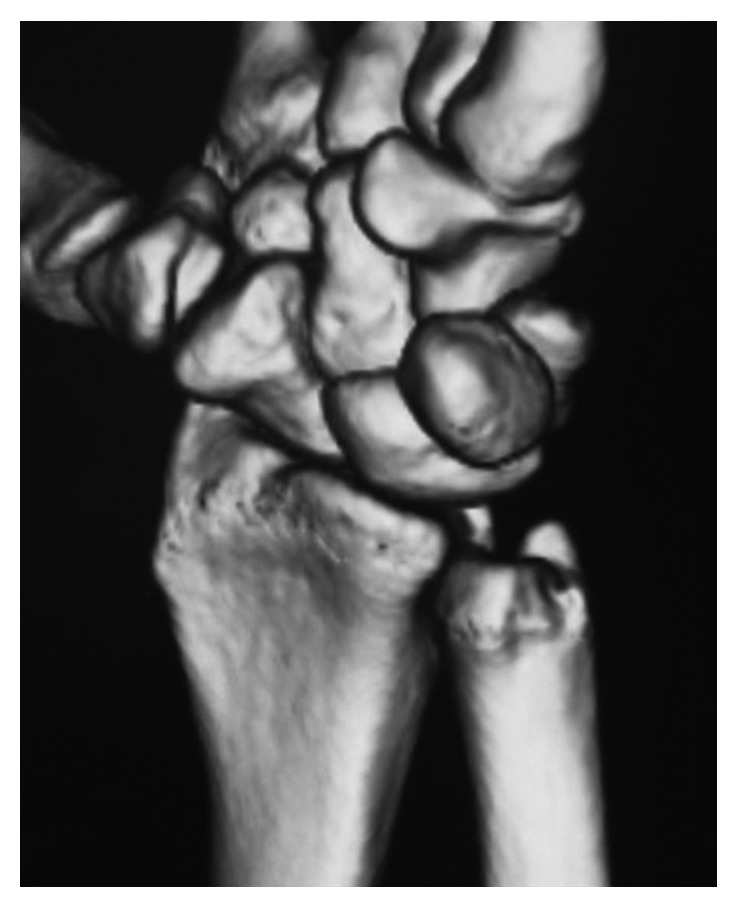
3D reconstruction of CT at 18 months postoperatively. It seemed the ulnar head had remodeled.

A 16-year-old boy sustained an injury when he fell while riding a bicycle. Physical examination showed swelling and tenderness on the ulnar side of his left wrist, and his pain worsened when he attempted to supinate his left forearm. Radiography and computed tomography (CT) in the supinated position revealed a dislocated ulnar head fracture, which was classified as Q4 in the AO fracture classification (Figures 1 and 2). Therefore, an operation was planned. Under brachial plexus block, we approached from the volar aspect in the supinated position (Figure 3) because we identified a bone fragment on the volar side of his left wrist in the preoperative CT taken in the supinated position. During surgery, we found the uncomminuted ulnar head fracture that was expected from the preoperative image. We temporarily fixed the bone fragment using a C-wire and attempted to fix it with DTJ Mini Screw® (Double Threaded Japan screw, Meira, Japan), which is a cannulated headless double-threaded screw. However, the bone fragment fractured when we inserted the screw. Because we were unable to achieve stabilization by using a screw, we resected the bone fragment. We found no obvious ligament around the resected fragment. After bone resection, we confirmed no obvious instability or disturbance of supination and pronation. After the operation, we followed the patient who did not require a splint or hand therapy. At the 18-month follow-up, his left grip strength was 44 kg. He recovered 80° of pronation and 90° of supination without supination pain. His right grip strength was 46 kg, and range of motion was the same with that of the left side (Figure 4). We could not find signs of triangular fibrocartilage complex (TFCC) injuries including the ulnar fovea sign and pain elicited by the ulnocarpal stress test. His DASH score was 0, and his final outcome was considered excellent (Figures 5 and 6).

## 3. Discussion

To the best of our knowledge, there are only four reports of pediatric distal ulnar epiphysiolysis without DRF [1–4], and there are only four previous reports of isolated ulnar head fracture in adults [5–8]. The present case might not be pediatric, because the growth plates are closed. Therefore, we discuss mainly previous 4 reports of adults. Jakab et al. [5] proposed that the injury mechanism was a direct blow to the ulnar aspect of the wrist in the supinated position of the forearm because in full supination, the ulnar head is almost completely uncovered, with contact occurring only at the volar aspect of the sigmoid notch. In contrast, Goikoetxea et al. [7] reported that the cause of the fracture was the combination of wrist extension, increased forearm pronation, and forced elbow flexion. They reported that at maximum pronation, the head of the ulna stays dorsally as the radius translates volarly, leaving only a small part of the ulna head articulating with the dorsal margin of the sigmoid fossa of the radius and with the ulna staying more dorsally in the elbow flexed position because pronation decreases with flexing of the elbow. They believed that the fracture occurred because the ulnar head was driven against the dorsal rim of the sigmoid notch in the position. In this case, the patient could have sustained the fracture in a position involving wrist extension, forearm pronation, and elbow flexion because he was probably holding the handlebars while riding the bicycle. The preoperative CT that showed the bone fragment of the ulnar head on the radial side combined with the supination pain suggested that the mechanism of injury reported by Goikoetxea et al. [7] was applicable to our case.

Two patients among four previous cases of isolated ulnar fractures were treated with cannulated compression screws, and one patient was treated by applying fixation with a Herbert screw. The patient outcomes in the three cases were almost good or excellent. The other case was conservatively managed with a wrist splint because obvious dislocation was not observed in the initial radiographs. However, radiographs at week 5 postinjury showed displacement with shortening and eventual malunion at 2 months. Although the patient regained normal function after hand therapy over 6 months, she reported some discomfort in the distal radioulnar joint [8].

The rationale for the surgery in our case was the bone fragment could cause the supination pain. Because the ulnar head fracture was an intra-articular fracture and the bone fragment was very small and very thin, we had to use a small headless screw. Acutrak 2 Micro® (Acumed, Hillsboro, OR), which was the smallest headless screw in Japan, needs drilling before inserting the screw. On the other hand, DTJ Mini Screw does not always need drilling before inserting a screw, and it was the second smallest headless screw in Japan. Therefore, we first attempted internal fixation with DTJ Mini Screw to avoid an intraoperative fracture while drilling. However, we ultimately had to resect the bone fragment because the screw caused additional fracturing. In the present case, from the preoperative images, we expected the bone fragment did not include the attachment of TFCC and we could not actually find obvious ligament around the bone fragment. Despite this unplanned bone resection, the patient had not suffered from obvious functional disorder, and there were no clear clinical signs of TFCC injuries during the 18-month follow-up. However, we do not advocate bone resection other than failure of fixation and the difficult case to perform internal fixation. Although longer follow-up would be needed because bone resection might lead to osteoarthritis of the distal radioulnar joint in the future, bone resection might be an option for isolated ulnar head fracture if a bone fragment is too small to allow performing internal fixation and does not have the attachment of TFCC.
